# Physical therapist perceptions and use of clinical pain mechanism assessment in the musculoskeletal setting: a survey analysis

**DOI:** 10.1186/s12891-023-06618-0

**Published:** 2023-06-22

**Authors:** Dhinu J. Jayaseelan, David A. Scalzitti, Carol A. Courtney

**Affiliations:** 1 Department of Health, Human Function, and Rehabilitation Sciences, The George Washington University, DC Washington, USA; 2 Department of Physical Therapy & Human Movement Sciences, Northwestern University, Chicago, IL USA

**Keywords:** Evaluation, Nociceptive, Neuropathic, Nociplastic, Quantitative sensory testing

## Abstract

**Background:**

A mechanism-based approach to the evaluation and management of pain has been suggested across disciplines in contemporary research. However, the translation of pain mechanism assessment strategies in research to clinical practice is unclear. This study sought to explore perceptions and use of clinical pain mechanism assessment by physical therapists managing musculoskeletal pain.

**Methods:**

This was an electronic cross-sectional survey. After initial development, refinement, and piloting for comprehensiveness, comprehensibility and relevance, the survey was disseminated to members of the Academy of Orthopaedic Physical Therapy via email listserv. Data was maintained anonymously using the online database REDCap. Descriptive statistics and Spearman’s correlations for non-parametric data were analyzed for frequencies and associations across variables.

**Results:**

In total, 148 respondents completed all aspects of the survey. Respondent age ranged from 26 to 73 years, with a mean (SD) of 43.9 (12.0). Most respondents (70.8%) reported performing clinical pain mechanism assessments at least ‘sometimes’. A majority (80.4%) believed clinical pain mechanism assessments are useful in guiding management strategies while 79.8% reported specifically choosing interventions to alter aberrant pain mechanisms. The most commonly used pain severity, physical examination testing and questionnaires were the numeric pain rating scale, pressure pain thresholds and pain diagrams, respectively. However, the vast majority of instruments to clinically assess pain mechanisms were performed by a small proportion of respondents (< 30%). There were no significant correlations between age, years of experience, highest earned degree, completion of advanced training or specialist certification and testing frequency.

**Conclusion:**

The evaluation of pain mechanisms involved in the pain experience is becoming common in research. The clinical application of pain mechanism assessment is unclear. Based on the results of this survey, physical therapists in the orthopedic setting believe pain mechanism assessment is useful, but data suggests it is infrequently performed. Additional research to uncover clinician motivation related to pain mechanism assessment is warranted.

**Supplementary Information:**

The online version contains supplementary material available at 10.1186/s12891-023-06618-0.

## Introduction

Musculoskeletal pain is a prevalent complaint. In 2016, an estimated $360.8 billion was spent in the United States on musculoskeletal disorders [1]. While there has been a substantial improvement in the understanding of pain, an individual’s pain experience remains personal and requires individualized care. Importantly, pain can exist in the absence of tissue damage, [2] making a strictly biomedical approach to pain management less relevant or effective in some cases. In fact, numerous environmental and contextual variables independent of the health condition itself play an important role in development and maintenance of chronic pain [3]. Considering the complexity of pain, it is important to understand how clinicians are currently evaluating pain in order to identify opportunities to improve practice.

One method in better understanding symptoms is to assess and identify the pain mechanism(s) involved in a person’s pain experience. By determining the predominant mechanism of nociceptive, neuropathic, or nociplastic pain, clinicians may be better equipped to formulate an appropriate and effective treatment plan [4, 5]. According to the International Association for the Study of Pain, nociceptive pain is defined as pain coming from actual or threatened damage to non-neural tissue whereas neuropathic pain is defined as pain caused by damage or dysfunction of the somatosensory nervous system [2]. Nociplastic pain on the other hand, which was more recently defined, refers to pain from altered nociception without clear evidence of actual or threatened tissue damage which would lead to the activation of peripheral nociceptors or damage to the nervous system [2]. Within these broad pain classifications are numerous physiological mechanisms that may affect neuroplasticity [6]. Various signs and symptoms found in patients with chronic pain may be due to distinct aberrant mechanisms [7]. However, while these definitions are widely used, pain is not always explicitly related to a single mechanism. Pain may often be related to multiple input or processing mechanisms, or a mixed presentation, making identification of the predominant mechanism involved challenging. For example, patellofemoral pain or tendinopathy which usually present as localized pain aggravated with movement tend to be classified as peripheral nociceptive pain. However, this peripheral nociceptive pain presentation may co-exist with aberrant central pain processing, possibly contributing to suboptimal long-term outcomes in some cases [8, 9]. In fact, when an international panel of experts was surveyed in a recent Delphi study, there was substantial overlap in which characteristics discriminated mechanism-based pain classifications, suggesting more work in this area is necessary [10].

Pain mechanism assessment is not new to research. For more than 20 years, pain has been evaluated using the mechanism-based concept grounded in the idea that clinical presentations reflect different pathophysiological mechanisms of pain generation [11]. Quantitative sensory testing (QST) is one modality to detect involved pain mechanisms, whereby sensory thresholds and stimulus tolerance are evaluated using patient feedback [10]. Abnormalities in thermal, vibratory, electrical and pressure thresholds may implicate peripheral and/or central mediators of an individual’s pain experience. Protocols have been created to attempt to standardize QST [12] although variability in testing procedures remains. While pain mechanism-based classifications are becoming commonly used in research, their application or relevance in clinical practice is unclear.

In physical therapy, a lack of time, a lack of skills, and misconceptions of evidence-based practice have been highlighted as common barriers to knowledge translation [13]. As it relates to physical therapist utilization of professional development tools for chronic pain, the primary barriers to using available tools were forgetting the resources were available or forgetting to use them [14]. Although attempts have been made to improve the clinical utility of pain mechanism assessments in physical therapy, [5, 15, 16] it is unknown if clinicians view these assessments as relevant, useful or feasible in practice. It is also unknown if certain perceptions or clinician characteristics are linked to pain mechanism assessment performance. As part of the early phases of the knowledge translation process in physical therapy, it is essential to assess knowledge, skills, attitudes and beliefs of clinicians, including contextual factors, that may play a role [17]. In this case, elucidating clinician views on the utility of clinical pain mechanism assessments and current practice tendencies can potentially guide research in knowledge translation strategies.

The purpose of this study was to evaluate perceptions and use of clinical pain mechanism assessment by physical therapists in the musculoskeletal setting.

## Methods

### Study design

This study was a cross-sectional survey. The study was approved by The George Washington University Institutional Review Board.

### Survey development

The survey was initially developed by two authors with substantial clinical and research experience in pain assessment and treatment (DJ, CC). To capture pertinent data, the instrument was divided into three sections: (1) perceptions of clinical pain mechanism assessment utility, (2) tendencies in clinical pain mechanism assessment and (3) respondent demographic information. To assess clinical assessment tendencies, ‘select all that apply’ questions identified tools used in practice, if any. If particular instruments were not listed as options, a free text box to fill in answers was provided. Likert-type questions explored respondent agreement to various questions or frequency of instrument utilization. Following initial questionnaire development, an external colleague with extensive expertise in survey methods was consulted and the instrument was modified according to their feedback. Afterwards, the survey was sent to a convenience sample of 20 randomly selected physical therapists from The George Washington University Hospital Outpatient Rehabilitation Center who consistently treat painful conditions to pilot test the instrument for relevance, comprehensiveness and comprehensibility. After relevant modifications were made, the final survey was reviewed and agreed upon by all authors. The final instrument can be found in the Appendix.

### Data Collection and Analysis

This study was completed with physical therapist respondents in the United States. Survey invitations were sent electronically through the Academy of Orthopaedic Physical Therapy in June 2022 with a reminder social media posting one week after initial dissemination. The survey remained open for 30 days through July 2022. Study data were collected and managed using the Research Electronic Data Capture (REDCap) website [18]. Informed consent was obtained electronically through participation and respondent data was collected anonymously. Only responses with 100% completion were included for analysis.

Data was analyzed using IBM SPSS Statistics for Windows, Version 28.0 (Armonk, NY: IBM Corp). Survey responses were primarily nominal or ordinal. Descriptive statistics were reported for frequencies and crosstabulations were performed as relevant to assess relationships between categorical variables. Correlational analysis was completed to identify relationships between demographic data, perceptions of pain mechanisms and frequency of assessment performance. Spearman’s rank correlations were performed to determine associations between these nonparametric variables. Significance levels were a priori set at *p* = 0.05.

## Results

### Participants

In total, 176 individuals opened the survey while 148 respondents finished all aspects of the survey, for a completion rate of 84.1%. Demographic information of respondents is presented in Table 1. Respondent age ranged from 26 to 73 years, with a mean (SD) of 43.9 (12.0). Professional experience was wide-ranging, with the largest proportions being between 0 and 5 and greater than 25 years. The majority noted a clinical doctorate as their highest earned degree (70.3%), while a relatively small proportion completed post-professional residency or fellowship training (23.0% and 12.2%, respectively). Most respondents (56.8%) reported being full-time clinicians, and 22.3% noted ‘other’ as a primary professional role, which typically indicated a combination of multiple roles (e.g. clinician and administrator). When asked about their patient pain presentations, most respondents noted a mixed patient population, rather than primarily having a patient population with predominant peripheral, spinal, chronic or widespread pain.

**Table 1:** Respondent demographic information

Variable	Response			N (%)		
Preferred pronouns	She/her			81 (54.7)		
	He/him			61 (41.2)		
	They/them			2 (1.4)		
	Other			4 (2.7)		
Years of experience	0–5			32 (21.6)		
	6–10			24 (16.2)		
	11–15			20 (13.5)		
	16–20			10 (6.8)		
	21–25			20 (13.5)		
	> 25			42 (28.4)		
Highest earned degree	Bachelor’s			5 (3.4)		
	Master’s			9 (6.1)		
	Clinical Doctorate (DPT)			104 (70.3)		
	Academic Doctorate (PhD, ScD, DSc, EdD, etc.)			30 (20.3)		
Completed residency training	No			114 (77.0)		
	Yes			34 (23.0)		
Completed fellowship training	No			130 (87.8)		
	Yes			18 (12.2)		
Board certified clinical specialist	No			61 (41.2)		
	Yes *(specialty below)*			87 (58.8)		
	Clinical Electrophysiology			1 (0.7)		
	Geriatrics			3 (2.0)		
	Orthopedics			83 (56.1)		
	Pediatrics			1 (0.7)		
	Sports			4 (2.7)		
Primary professional role	Full-time administrator			2 (1.4)		
	Full-time clinician			84 (56.8)		
	Full-time educator			26 (17.6)		
	Full-time researcher			3 (2.0)		
	Other			33 (22.3)		
***Patient Demographics Reported by Respondents***						
Predominant type of pain:	*0–25%*	*26–50%*	*51–75%*		*76–100%*	
Peripheral pain	24 (16.2)	69 (46.6)	40 (27.0)		15 (10.1)	
Spinal pain	11 (7.4)	59 (39.9)	63 (42.6)		15 (10.1)	
Chronic pain	27 (18.2)	68 (45.9)	31 (20.9)		22 (14.9)	
Widespread pain	99 (66.9)	40 (27.0)	7 (4.7)		2 (1.4)	

### Clinical pain mechanism assessment perceptions

Respondent perceptions related to clinical pain mechanism assessment are presented in Table 2. When asked how frequently they perform clinical pain mechanism assessment, 70.8% reported identifying predominant pain mechanisms as least ‘*sometimes’*. However, when asked if pain mechanism assessment is reserved for research settings, 66.9% disagreed, with a similar proportion (67.6%) agreeing that clinical pain mechanism assessment is feasible. A majority (80.4%) of respondents agreed that pain mechanism assessment is useful to guide management strategies, and 79.7% agreed that they specifically select interventions to alter aberrant pain mechanisms. Respondents were also queried about potential barriers to clinical pain mechanism assessments. A number of respondents agreed that they would perform pain mechanism assessments more frequently if tools were less expensive (44%), if tools have good diagnostic utility (56.1%), or if tools were less time consuming (46.6%); however, the largest proportion of respondents remained neutral in answering each of these questions. Responses were similarly distributed when asked about proficiency with pain mechanism assessments.

**Table 2:** Respondent perceptions of clinical pain mechanism assessment

Survey Item	N (%)				
	Never	Almost Never	Sometimes	Often	Always
*How often do you incorporate testing to identify the predominant pain mechanism(s) involved in a patient’s pain experience?*	17 (11.5)	26 (17.6)	27 (18.2)	43 (29.1)	35 (23.6)
	**Strongly Disagree**	**Somewhat Disagree**	**Neutral**	**Somewhat Agree**	**Strongly Agree**
*In my practice, pain mechanism assessment is reserved for research or laboratory settings.*	76 (51.4)	23 (15.5)	22 (14.9)	19 (12.8)	8 (5.4)
*In my practice, clinical pain mechanism assessment is useful for guiding management strategies.*	2 (1.4)	7 (4.7)	20 (13.5)	50 (33.8)	69 (46.6)
*In my practice I purposely select an intervention for a patient to alter a specific aberrant pain mechanism.*	6 (4.1)	9 (6.1)	15 (10.1)	57 (38.5)	61 (41.2)
*In my practice, clinical pain mechanism assessment is feasible.*	7 (4.7)	13 (8.8)	28 (18.9)	58 (39.2)	42 (28.4)
*If pain mechanism assessment tools were less expensive, I would use them more frequently.*	13 (8.8)	11 (7.4)	59 (39.9)	39 (26.4)	26 (17.6)
*If pain mechanism assessment tools had good diagnostic utility, I would use them more frequently.*	11 (7.4)	10 (6.8)	44 (29.7)	43 (29.1)	40 (27.0)
*If pain mechanism assessment tools were not so time consuming, I would use them more frequently.*	10 (6.8)	16 (10.8)	53 (35.8)	37 (25.0)	32 (21.6)
*I think pain mechanism assessment tools could be useful, but I do not know how to use them.*	38 (25.7)	25 (16.9)	29 (19.9)	29 (19.9)	27 (18.2)

### Clinical pain mechanism assessment instrument usage

**Fig. 1:**
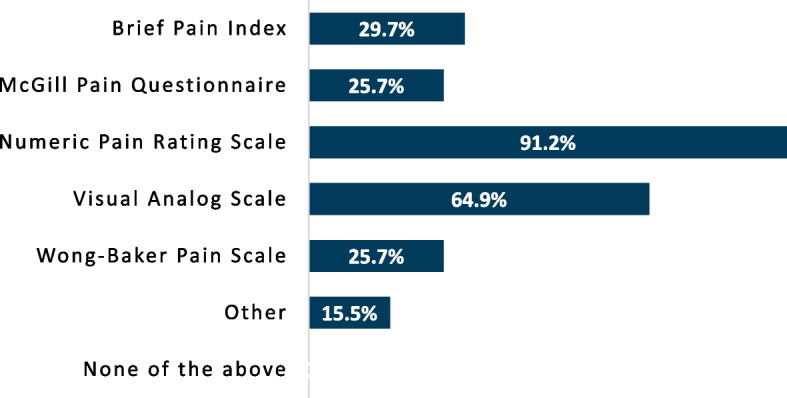
Proportion of respondents using individual instruments to evaluate pain severity

**Fig. 2:**
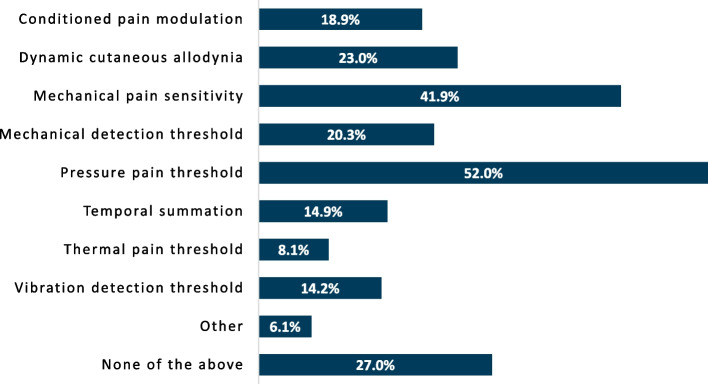
Proportion of respondents using individual physical examination instruments

**Fig. 3:**
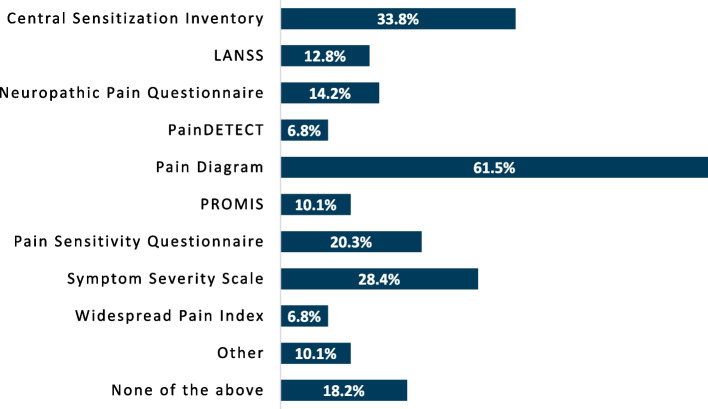
Proportion of respondents using individual questionnaires to detect pain mechanisms

**Fig. 4:**
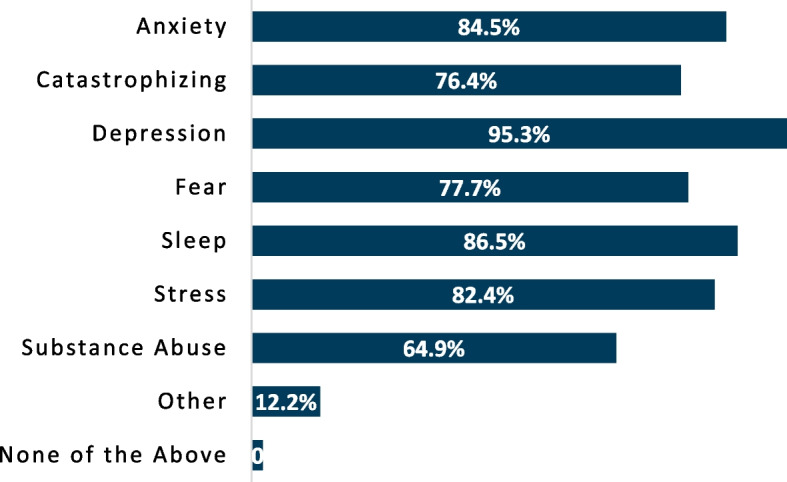
Proportion of respondents assessing individual contributing variables associated with pain

The percentage of respondents using a given instrument was collected and analyzed. When asked which tools are employed to assess pain severity (Fig. 1), the numeric pain rating scale was the most commonly incorporated, with 91.2% of respondents using the instrument. Physical testing to evaluate pain mechanisms appeared to be infrequently performed (Fig. 2), but when done, respondents most commonly assessed pressure pain threshold (52.0%). Beyond pressure pain thresholds and cutaneous mechanical pain sensitivity (41.9%), no physical examination instrument was performed by more than 25% of respondents, with 27.0% reporting not using any physical testing tools. A pain diagram was the most commonly used questionnaire by respondents (61.5%) whereas other questionnaires were infrequently used to assess predominant pain mechanisms involved (Fig. 3). Aside from pain diagrams and the central sensitization inventory (33.8%) no questionnaires were used by ≥ 30% of respondents. Comparatively, numerous variables potentially contributing to pain (e.g. anxiety, depression, sleep, etc.) were assessed at a higher proportion, with nearly two-thirds of respondents reporting investigation of each item in their practice (Fig. 4). Specific frequency of individual instrument usage to assess the pain experience was also analyzed, and is presented in Table 3.

**Table 3:** Frequency of clinical pain mechanism assessment instrument utilization

INSTRUMENT	RESPONSE (N (%))				
	NEVER	ALMOST NEVER	SOMETIMES	OFTEN	ALWAYS
***Pain severity***					
Brief Pain Index	73 (49.3)	17 (11.5)	24 (16.2)	24 (16.2)	10 (6.8)
McGill Pain Questionnaire	76 (51.4)	29 (19.6)	23 (15.5)	19 (12.8)	1 (0.7)
Numeric Pain Rating Scale	4 (2.7)	1 (0.7)	7 (4.7)	40 (27.0)	96 (64.9)
Visual Analog Scale	22 (14.9)	11 (7.4)	36 (24.3)	29 (19.6)	50 (33.8)
Wong-Baker Pain Scale	71 (48.0)	27 (18.2)	36 (24.3)	8 (5.4)	6 (4.1)
Other	60 (40.5)	14 (9.5)	44 (29.7)	16 (10.8)	14 (9.5)
***Physical examination***					
Conditioned Pain Modulation	90 (60.8)	20 (13.5)	20 (13.5)	15 (10.1)	3 (2.0)
Cutaneous Mechanical Pain Sensitivity	61 (41.2)	15 (10.1)	35 (23.6)	30 (20.3)	7 (4.7)
Dynamic Mechanical Cutaneous Allodynia	84 (56.8)	20 (13.5)	26 (17.6)	16 (10.8)	2 (1.4)
Mechanical Detection Threshold	78 (52.7)	23 (15.5)	28 (18.9)	14 (9.5)	5 (3.4)
Pressure Pain Threshold	55 (37.2)	15 (10.1)	32 (21.6)	39 (26.4)	7 (4.7)
Temporal Summation	91 (61.5)	33 (22.3)	16 (10.8)	7 (4.7)	1 (0.7)
Thermal Pain Threshold	104 (70.3)	27 (18.2)	12 (8.1)	4 (2.7)	1 (0.7)
Vibration Detection Threshold	94 (63.5)	23 (15.5)	27 (18.2)	2 (1.4)	2 (1.4)
Other	90 (60.8)	18 (12.2)	25 (16.9)	9 (6.1)	6 (4.1)
***Pain mechanism questionnaires***					
Central Sensitization Inventory	85 (57.4)	15 (10.1)	22 (14.9)	23 (15.5)	3 (2.0)
Leeds Assessment of Neuropathic Symptoms and Signs (LANSS)	118 (79.7)	13 (8.8)	7 (4.7)	10 (6.8)	0 (0)
Neuropathic Pain Questionnaire	103 (69.6)	19 (12.8)	17 (11.5)	8 (5.4)	1 (0.7)
PainDETECT	123 (83.1)	13 (8.8)	8 (5.4)	3 (2.0)	1 (0.7)
Pain Diagram	25 (16.9)	11 (7.4)	21 (14.2)	35 (23.6)	56 (37.8)
Patient-Reported Outcomes Measurement Information System (PROMIS) Pain Interference	89 (60.1)	23 (15.5)	16 (10.8)	11 (7.4)	9 (6.1)
Pain Sensitivity Questionnaire	98 (66.2)	12 (15.5)	14 (9.5)	10 (6.8)	3 (2.0)
Symptom Severity Scale	77 (52.0)	18 (12.2)	26 (17.6)	18 (12.2)	9 (6.1)
Widespread Pain Index	122 (82.4)	16 (10.8)	9 (6.1)	1 (0.7)	0 (0)
Other	96 (64.9)	19 (12.8)	16 (10.8)	10 (6.8)	7 (4.7)
***Possible contributing variables***					
Anxiety	7 (4.7)	8 (5.4)	25 (16.9)	55 (37.2)	53 (35.8)
Catastrophizing	13 (8.8)	10 (6.8)	37 (25.0)	53 (35.8)	35 (23.6)
Depression	2 (1.4)	2 (1.4)	25 (16.9)	54 (36.5)	65 (43.9)
Fear	11 (7.4)	4 (2.7)	31 (20.9)	63 (42.6)	39 (26.4)
Sleep	8 (5.4)	4 (2.7)	22 (14.9)	46 (31.1)	68 (45.9)
Stress	8 (5.4)	2 (1.4)	29 (19.6)	49 (33.1)	60 (40.5)
Substance Abuse	16 (10.8)	23 (15.5)	37 (25.0)	35 (23.6)	37 (25.0)
Other	65 (43.9)	11 (7.4)	46 (31.1)	12 (8.1)	14 (9.5)

### Relationships between assessed variables

To determine possible associations among respondent perceptions, practice, and demographics, correlation testing was competed. Given the non-parametric data, Spearman’s rank correlation analysis was performed to determine associations between variables (Table 4). The reported frequency of clinical pain mechanism assessment was negatively correlated to the belief that testing is performed only for research (*ρ* = -0.698, *p* < 0.001), and positively correlated to perceived usefulness of assessment to guide management (*ρ* = 0.663, *p* < 0.001) and assessing pain mechanisms with the intent of targeting specific mechanisms (*ρ* = 0.576, *p* < 0.001). Other moderate to strong correlations were seen among perceptions related to testing. However, when assessing correlations between testing frequency or perceptions and demographic data, few relationships were found. Specifically, there were no significant correlations between age, years of experience, highest earned degree, completion of advanced training or specialist certification and testing frequency. While some correlations between included variables were determined to be statistically significant (*p* < 0.05), relationships were weak or very weak and therefore not deemed influential.

**Table 4:** Spearman’s rank correlation coefficient of testing perceptions and demographics

	Testing Frequency	Testing is for Research Only	Testing is Useful for Management	I Use Testing for Management
**Testing Frequency**	1.000	-0.698^b^	0.663^b^	0.576^b^
**Testing for Research Only**	-0.698^b^	1.000	-0.707^b^	-0.584^b^
**Testing is Useful for Management**	0.663^b^	-0.707^b^	1.000	0.593^b^
**I Use Testing for Management**	0.576^b^	-0.584^b^	0.593^b^	1.000
**Years Old**	0.057	-0.153	0.094	0.150
**Years of Experience**	0.090	-0.179a	0.085	0.191^a^
**Highest Earned Degree**	0.076	0.059	-0.078	-0.099
**Completed Residency Training**	0.024	0.038	-0.043	0.011
**Completed Fellowship Training**	0.127	-0.027	-0.014	0.167^a^
**Clinical Specialist**	0.075	-0.145	0.019	0.120
**Percentage of Predominant Peripheral Pain**	-0.201^a^	0.102	-0.149	-0.134
**Percentage of Predominant Spinal Pain**	0.113	-0.117	0.107	0.177^a^
**Percentage of Chronic Pain**	0.157	-0.115	0.159	0.171^a^
**Percentage of Widespread Pain**	0.161^a^	-0.123	0.127	0.161

^a^Correlation is significant at the 0.05 level (2-tailed)

^b^Correlation is significant at the 0.01 level (2-tailed)

## Discussion

This study sought to examine the perceptions and clinical use of clinical pain mechanism assessments among physical therapists. Based on the results, the majority of respondents appear to believe that pain mechanism assessment is useful, feasible, and can help guide management strategies. Despite these beliefs, physical examination and questionnaire instruments developed to support mechanism-based classifications appear to be infrequently used in the clinical setting.

Quantitative sensory testing (QST) can be a reliable pain assessment tool used in research studies [19–21]. When examining potential barriers to clinical pain mechanism assessments, some respondents noted expense, time, and diagnostic utility as potential factors. While sophisticated research equipment can be expensive, inexpensive methodology options for detecting aberrant central pain processing exist and have been previously suggested [15]. Additionally, handheld dynamometry, typically used to assess force output, can be a valid and reliable assessment of pressure pain thresholds [16] and QST may also discriminate severity categories on pain questionnaires [22]. These findings suggests clinically relevant equipment may be already available and could serve multiple purposes. Efficiency of testing is relevant, and tests considered laborious may be less frequently used. Interestingly, some QST perceived to be time consuming, e.g. conditioned pain modulation or temporal summation, have protocols that can be completed effectively within minutes, [23, 24] but additional testing in various populations could confirm findings. Physical examination testing, although infrequently performed according to data from respondents in this survey, can be done quickly, reliably and cost-effectively.

Physical therapists also infrequently use appropriate written instruments to assess pain. Pain diagrams were reported as the most commonly used questionnaire among the respondents. Pain diagrams and mapping can be helpful in identifying subgrouping and differentiating pain mechanisms [25–27]. It is unclear however, if respondents use pain diagrams specifically for the purpose of differentiating pain mechanisms, or primarily as part of an intake to assist with clinical pattern recognition of various health conditions. Aside from pain diagrams and the central sensitization inventory, respondents did not regularly use questionnaires as part of their clinical pain mechanism assessment. As previously noted, 44% and 51.6% of respondents suggested they would use tools more frequently if they were less expensive and had good diagnostic utility, respectively. Interestingly, some of the freely available tools offered as options in the survey have demonstrated sufficient diagnostic utility and predictive value [28, 29]. While questionnaires may not independently identify predominant pain mechanisms and should not replace comprehensive clinical examinations, they may be useful in classifying individuals using a mechanisms-based approach.

As compared to the infrequent physical testing and questionnaire utilization, according to the data from the respondents included in this sample, variables potentially contributing to pain (e.g. anxiety, depression, etc.) were more frequently assessed. The findings of this study appear consistent with a cross-sectional survey exploring psychosocial practice in Australia, where respondents frequently reported confidence and proficiency in psychosocial skills [30]. However, Man et al. noted inconsistency in practice in their study, potentially reflecting a discrepancy in how psychosocial proficiency is defined and how psychosocial dysfunction is managed [30]. A take away from their study could be that many clinicians assess psychosocial variables but it is unclear if these variables are accurately or appropriately managed. While proportionally more respondents from this survey reported assessing contributing psychosocial variables, it is somewhat surprising that these items are not more commonly assessed. As pain is always a personal experience, it could be argued that understanding psychological and social influences on pain should occur consistently and lead to multimodal and multidisciplinary care as needed.

One could posit that the clinical use, or lack thereof, of pain mechanism assessment is dependent on one’s educational training. For example, if an individual did not learn about clinical pain mechanisms or QST in their didactic or clinical training, they would be unlikely to use QST to detect predominant mechanisms. A pain education manual recently developed by the Academy of Orthopaedic Physical Therapy supports the incorporation of pain mechanism-based content into physical therapist professional degree programs [31]. According to a recent survey analysis of accredited physical therapy programs in the United States, pain is delivered in non-uniform methods and only 63% of respondents reported using QST in their curriculum [32]. Interestingly, based on findings from this study, there was no correlation between level of education, experience or training and testing frequency. It is possible clinicians may be aware of pain mechanisms-based evaluation and management evidence, but have a low usage rate based not on awareness but rather their patient population. For example, those treating patellar of Achilles tendinopathy may feel less inclined to use QST since these are considered primarily peripheral nociceptive pain states, as compared to rotator cuff or lateral elbow tendinopathy, which may be more associated with nociplastic pain [9].

While clinical pain mechanism assessment may assist in discriminating predominant mechanisms involved in a patient’s presentation, its utility in guiding effective management strategies requires additional investigation. There has been some evidence of a correlation between QST findings and analgesic responses, although the relationship requires further inquiry [33, 34]. Several studies have demonstrated that physical therapy interventions such as transcutaneous electrical stimulation, [35] exercise [36] and manual therapy [37] facilitate descending inhibition of pain as measured by the very simple QST measure of conditioned pain modulation, however this QST appears to be not prevalent in physical therapy clinical practice. Interestingly, Petersen et al. suggest that conditioned pain modulation and temporal summation show the most consistent predictive values for chronic postoperative pain and analgesic effect [38]. From a theoretical perspective, matching interventions to the involved pain mechanism(s) makes sense, however it is unclear how specifically physical therapy interventions target a given mechanism. With a potential shift towards precision medicine which tailors intervention strategies to patient specific factors, clinical pain mechanism assessments seem to be a rationale, non-invasive and feasible approach.

This study comes with a number of limitations. With surveys it is important to consider data may be impacted by sampling and response bias. Sampling was completed through listserv distribution of a single professional organization. While those who received the email invitation had no responsibility to complete the survey, it is possible that those who responded have a particular interest in the topic. Also, it was not feasible to calculate the response rate, as it is unclear how many of those receiving the email invitation actually engaged with the survey to respond or not respond (e.g., many may have deleted without reading). While a completion rate was calculated, having a response rate would increase the confidence in the results. The findings from this sample should be interpreted with caution as they may not completely reflect the population’s clinical practice tendencies and perceptions, although the sample’s demographic data does loosely match that of the professional association invited to participate. Similarly, the scope of this study is limited to observational analysis of use and perception of clinical pain mechanism assessments. Few judgements can be made related to the management strategies of clinicians based on various mechanism-based presentations. However, as there is currently no evidence reporting on clinical pain mechanism assessment practice, this could be considered a relevant first step in the knowledge translation process.

## Conclusion

One method of evaluating an individual’s pain experience is to investigate the predominant pain mechanisms involved. This survey sought to examine the perceptions of physical therapists regarding the use of clinical pain mechanisms assessments. Based on the findings, it appears the majority of respondents find value in using clinical pain mechanisms assessments in practice, although instruments to do so are infrequently used. Although the usefulness of ‘mechanism-specific’ interventions requires more research, it is possible that outcomes in pain management could be enhanced by understanding predominant mechanisms involved. To this end, identifying ways to improve the clinical evaluation of pain mechanisms could be useful. Continued investigation into motivation to use or not use clinical pain mechanism assessments, and their relevance to positive treatment outcomes, is warranted to facilitate knowledge translation of this content.

## Supplementary Information

**Additional file 1.** 

## Data Availability

The datasets used and/or analyzed during the current study available from the corresponding author on reasonable request.

## References

[1] Dieleman JL, Cao J, Chapin A (2020). US Health Care spending by Payer and Health Condition, 1996–2016. JAMA.

[2] International Association for the Study of Pain. IASP Taxonomy. https://www.iasp-pain.org/Taxonomy. Updated 2012. Accessed 6/20, 2017.

[3] Courtney CA, Fernandez-de-Las-Penas C, Bond S (2017). Mechanisms of chronic pain - key considerations for appropriate physical therapy management. J Man Manip Ther.

[4] Vardeh D, Mannion RJ, Woolf CJ (2016). Toward a mechanism-based Approach to Pain diagnosis. J Pain.

[5] Chimenti RL, Frey-Law LA, Sluka KA (2018). A mechanism-based Approach to Physical Therapist Management of Pain. Phys Ther.

[6] Melzack R, Coderre TJ, Katz J, Vaccarino AL (2001). Central Neuroplasticity and Pathological Pain. Ann N Y Acad Sci.

[7] Lluch E, Nijs J, Courtney CA (2018). Clinical descriptors for the recognition of central sensitization pain in patients with knee osteoarthritis. Disabil Rehabil.

[8] Silva DDO, Rathleff MS, Petersen K, Azevedo F, Barton CJ (2019). Manifestations of Pain Sensitization Across different painful knee Disorders: a systematic review including Meta-analysis and metaregression. Pain Med.

[9] Rio E, Sandler J, Cheng K, Moseley GL, Cook J, Girdwood M (2021). Sensory Processing in People with and without Tendinopathy: a systematic review with Meta-analysis of local, Regional, and Remote Sites in Upper- and lower-limb conditions. J Orthop Sports Phys Ther.

[10] Shraim MA, Sluka KA, Sterling M (2022). Features and methods to discriminate between mechanism-based categories of pain experienced in the musculoskeletal system: a Delphi expert consensus study. Pain.

[11] Greenspan JD (2001). Quantitative assessment of neuropathic pain. Curr Pain Headache Rep.

[12] Rolke R, Baron R, Maier C (2006). Quantitative sensory testing in the German Research Network on Neuropathic Pain (DFNS): standardized protocol and reference values. Pain.

[13] Scurlock-Evans L, Upton P, Upton D (2014). Evidence-based practice in physiotherapy: a systematic review of barriers, enablers and interventions. Physiotherapy.

[14] Etheridge T, Bostick GP, Hoens AM, et al. Barriers to physiotherapists’ use of Professional Development Tools for Chronic Pain: a knowledge translation study. Physiotherapy Can. 2022;e20200148. 10.3138/ptc-2020-0148. 10.3138/ptc-2020-0148 PMC10262724 37324608

[15] van Griensven H, Schmid A, Trendafilova T, Low M (2020). Central Sensitization in Musculoskeletal Pain: lost in translation?. J Orthop Sports Phys Ther.

[16] Jayaseelan DJ, Cole KR, Courtney CA (2020). Hand-held dynamometer to measure pressure pain thresholds: a double-blinded reliability and validity study. Musculoskelet Sci Pract..

[17] Zidarov D, Thomas A, Poissant L (2013). Knowledge translation in physical therapy: from theory to practice. Disabil Rehabil.

[18] Harris PA, Taylor R, Thielke R, Payne J, Gonzalez N, Conde JG (2009). Research electronic data capture (REDCap)--a metadata-driven methodology and workflow process for providing translational research informatics support. J Biomed Inform.

[19] Rolke R, Magerl W, Campbell KA (2006). Quantitative sensory testing: a comprehensive protocol for clinical trials. Eur J Pain.

[20] Uddin Z, MacDermid JC (2016). Quantitative sensory testing in Chronic Musculoskeletal Pain. Pain Med.

[21] Nuwailati R, Bobos P, Drangsholt M, Curatolo M (2022). Reliability of conditioned pain modulation in healthy individuals and chronic pain patients: a systematic review and meta-analysis. Scand J Pain.

[22] Zafereo J, Wang-Price S, Kandil E (2021). Quantitative sensory testing discriminates Central Sensitization Inventory Scores in participants with Chronic Musculoskeletal Pain: an exploratory study. Pain Pract.

[23] Mackey IG, Dixon EA, Johnson K, Kong JT (2017). Dynamic quantitative sensory testing to Characterize Central Pain Processing. J Vis Exp.

[24] Arendt-Nielsen L, Larsen JB, Rasmussen S, Krogh M, Borg L, Madeleine P (2020). A novel clinical applicable bed-side tool for assessing conditioning pain modulation: proof-of-concept. Scand J Pain.

[25] Lluch Girbes E, Duenas L, Barbero M (2016). Expanded distribution of Pain as a sign of Central Sensitization in individuals with symptomatic knee osteoarthritis. Phys Ther.

[26] Alter BJ, Anderson NP, Gillman AG, Yin Q, Jeong JH, Wasan AD (2021). Hierarchical clustering by patient-reported pain distribution alone identifies distinct chronic pain subgroups differing by pain intensity, quality, and clinical outcomes. PLoS ONE.

[27] Sehgal N, Gordon DB, Hetzel S, Backonja MM (2021). Colored Pain drawing as a clinical Tool in differentiating Neuropathic Pain from Non-Neuropathic Pain. Pain Med.

[28] Scerbo T, Colasurdo J, Dunn S, Unger J, Nijs J, Cook C (2018). Measurement properties of the central sensitization inventory: a systematic review. Pain Pract.

[29] Mathieson S, Maher CG, Terwee CB, Folly de Campos T, Lin CW (2015). Neuropathic pain screening questionnaires have limited measurement properties. A systematic review. J Clin Epidemiol.

[30] Man I, Kumar S, Jones M, Edwards I (2019). An exploration of psychosocial practice within private practice musculoskeletal physiotherapy: a cross-sectional survey. Musculoskelet Sci Pract.

[31] Shepherd M, Courtney CA, Wassinger CA, Davis DS, Rubine B. Pain Education Manual For Physical Therapist Professional Degree Programs. Academy of Orthopedic Physical Therapy-Pain Special Interest Group. 2022. https://www.orthopt.org/uploads/content_files/files/Pain_Manual_Draft_FINAL_6.25.2021%281%29.pdf.

[32] Emerson AJ, Courtney CA, Alcon C, Shaffer SM. The current state of Pain Curricula in CAPTE Accredited Doctor of Physical Therapy Programs: a 2021 report. Pain Med. 2022;pnac145. 10.1093/pm/pnac145. 10.1093/pm/pnac145 36165701

[33] Grosen K, Fischer IW, Olesen AE, Drewes AM (2013). Can quantitative sensory testing predict responses to analgesic treatment?. Eur J Pain.

[34] Woolf CJ, Max MB (2001). Mechanism-based Pain diagnosis: issues for analgesic Drug Development. Anesthesiology.

[35] Dailey DL, Rakel BA, Vance CGT (2013). Transcutaneous electrical nerve stimulation reduces pain, fatigue and hyperalgesia while restoring central inhibition in primary fibromyalgia. Pain.

[36] Vaegter HB, Jones MD (2020). Exercise-induced hypoalgesia after acute and regular exercise: experimental and clinical manifestations and possible mechanisms in individuals with and without pain. Pain Rep.

[37] Courtney CA, Steffen AD, Fernandez-de-Las-Penas C, Kim J, Chmell SJ. Joint mobilization enhances mechanisms of Conditioned Pain Modulation in individuals with osteoarthritis of the knee. J Orthop Sports Phys Ther. 2016;1–30. 10.2519/jospt.2016.6259. 10.2519/jospt.2016.6259 26721229

[38] Petersen KK, Vaegter HB, Stubhaug A (2021). The predictive value of quantitative sensory testing: a systematic review on chronic postoperative pain and the analgesic effect of pharmacological therapies in patients with chronic pain. Pain.

