# Supplementary material for: Physical therapist perceptions and use of clinical pain mechanism assessment in the musculoskeletal setting: a survey analysis
Source: BMC Musculoskelet Disord. 2023 Jun 22;24:509. doi: 10.1186/s12891-023-06618-0 (PMC10286424; doi:10.1186/s12891-023-06618-0)
Supplement: Supplementary file 1 — Additional file 1. [file 12891_2023_6618_MOESM1_ESM.pdf]

Clinical Pain Mechanisms Assessment Survey

You are being asked to participate in this survey study because you are a member of the Academy of Orthopaedic Physical Therapy. The purpose of this survey study is to understand current practice trends in clinical pain mechanism assessment among physical therapists treating musculoskeletal pain in the United States. Your participation in this study is completely voluntary and your data will remain anonymous. Choosing to participate or abstaining from participating in this study will not negatively impact your current role or professional status. There is no financial renumeration for your participation. We will use the synthesized results to help inform doctoral, post-professional and continuing education programs where pain evaluation and management is taught.

This survey is separated into 3 separate sections: 1) perceptions related to clinical pain mechanisms assessments, 2) tools used in pain mechanism assessments, 3) your demographic information. Total time for completion should be less than 20 minutes. But again, no personal identifying information will be gathered and your responses are completely anonymous.

The study has been approved by The George Washington University, IRB #: NCR224156. The research team consists of:

Dhinu J. Jayaseelan, DPT, OCS, FAAOMPT, The George Washington University, Principal investigator

David A. Scalzitti, PT, PhD, The George Washington University, Co-investigator

Carol A. Courtney, PT, PhD, FAAOMPT, ATC, Northwestern University, Co-investigator

Should you have any questions please reach out directly to the principal investigator via email at dhinuj@gwu.edu or phone at (202) 994-5495.

Informed Consent

Informed Consent for Participation in a Research Study

Title of Study: Clinical pain mechanism assessment by physical therapists in the United States: a cross-sectional survey analysis.
IRB #: NCR224156
Principal Investigator Name: Dhinu J. Jayaseelan, DPT, OCS, FAAOMPT

You are invited to participate in a research study under the direction of Dr. Dhinu Jayaseelan of the Department of Health, Human Function, and Rehabilitation Sciences at The George Washington University (GWU). Taking part in this research is entirely voluntary. Your academic standing or the status of your employment will not, in any way, be affected should you choose not to participate or if you decide to withdraw from the study at any time. Further information regarding this study may be obtained by contacting Dhinu Jayaseelan (principal investigator), at telephone number (202) 994-5495 or via e-mail at dhinuj@gwu.edu.

The purpose of this study is to understand current trends, beliefs and barriers related to clinical pain mechanism assessment by physical therapists.

What are the reasons you might choose to volunteer for this study? You may choose to participate because you have an interest in pain evaluation and management, you are interested in contributing to the advancement of research, or a variety of personal reasons.

What are the reasons you might not choose to volunteer for this study?
You may choose not to participate in this study for a number of reasons. Primarily, it may be that you do not have the time or interest in participation. This topic of investigation may not be within your area of expertise. There is no financial or professional renumeration for participating in this study.

If you choose to take part in this study, you will complete an online survey. The survey consists of questions regarding your perceptions and utilization of clinical pain mechanism assessments as well as capturing demographic information. The total amount of time you will spend in connection with this study is approximately 20 minutes. You may stop your participation in this study at any time.

Possible risks or discomforts you could experience during this study are limited, but may include a loss of anonymity. Every attempt will be made during the survey creation to facilitate anonymity, and no personal identifying information will be collected. As this survey is completed electronically, it is possible that shared computers may allow other individuals to see your responses.

You will not benefit directly from your participation in the study. The benefits to science and humankind that might result from this study include a greater understanding of pain evaluation strategies currently employed by physical therapists. Gained information may help to inform knowledge translation from 'bench to bedside'.

Every effort will be made to keep your information confidential, however, this can not be guaranteed. None of the 18 personal identification information items established by HIPAA are being asked for. Within the secured online survey system RedCAP, all responses have been set to not require any personal information, and each item response is set to maintain anonymity. If results of this research study are reported in journals or at scientific meetings, the people who participated in this study will not be named or identified.

The Office of Human Research of George Washington University, at telephone number (202) 994-2715, can provide further information about your rights as a research participant.

Your willingness to participate in this research study is implied if you proceed.
*Please keep a copy of this document (attached) in case you want to read it again.

[Attachment: "Informed Consent - PainMechSur - Approved.pdf"]

Part 1. Perceptions related to clinical pain mechanism assessment

For the following questions, the phrase 'pain mechanism' refers to the biological pain mechanisms associated with the categories of nociceptive, neuropathic or nociplastic as described by the International Association for the Study of Pain (IASP). Some examples of aberrant pain mechanisms include the following:

With nociceptive pain, nociceptors that innervate the injured tissue are exposed to inflammatory mediators, resulting in decreased threshold to elicit a response from and hyperexcitability of these first order neurons. The result is primary hyperalgesia at the site of the injured tissue.

With neuropathic pain, transient receptor potential vanilloid channel 1, a nonselective cation channel, (TRPV1) receptors are up-regulated on neurons, causing reduced stimulation thresholds and thermal sensitivity, especially to heat.

With nociplastic pain, a variety of aberrant pain mechanisms may be found. One example is heterosynaptic facilitation which results clinically in a widening distribution of pain, and is referred to as secondary hyperalgesia. This can be regional, such as throughout a limb, or widespread. A second example is an impairment in the functionality of descending inhibitory pathways, often found in chronic pain populations.

How often do you incorporate testing to identify the predominant pain mechanism(s) involved in a patient's pain experience?

- ◯ Never
- ◯ Almost never
- ◯ Sometimes
- ◯ Often
- ◯ Always

For each of the following statements, please select the option with which you agree most.

	STRONGLY DISAGREE	SOMEWHAT DISAGREE	NEUTRAL	SOMEWHAT AGREE	STRONGLY AGREE
In my practice, pain mechanism assessment is reserved for research or laboratory settings.	○	○	○	○	○
In my practice, clinical pain mechanism assessment is useful for guiding management strategies.	○	○	○	○	○
In my practice I purposely select an intervention for a patient to alter a specific aberrant pain mechanism.	○	○	○	○	○
In my practice, clinical pain mechanism assessment is feasible.	○	○	○	○	○
If pain mechanism assessment tools were less expensive, I would use them more frequently.	○	○	○	○	○
If pain mechanism assessment tools had good diagnostic utility, I would use them more frequently.	○	○	○	○	○
If pain mechanism assessment tools were not so time consuming, I would use them more frequently.	○	○	○	○	○
I think pain mechanism assessment tools could be useful, but I do not know how to use them.	○	○	○	○	○

Part 2. Tools used to evaluate predominant pain mechanisms.

Which of the following tools do you use in clinical assessment of pain severity? (Select ALL that apply)

- ☐ Brief pain inventory
- ☐ McGill pain questionnaire
- ☐ Numeric pain rating scale (NPRS)
- ☐ Visual analog scale (VAS)
- ☐ Wong-Baker faces pain scale
- ☐ Other
- ☐ None of the above

Please list what other tools you use to measure pain severity.

____

How frequently do you use the following pain severity assessment tools?

	NEVER	ALMOST NEVER	SOMETIMES	OFTEN	ALWAYS
Brief pain inventory	○	○	○	○	○
McGill pain questionnaire	○	○	○	○	○
Numeric pain rating scale (NPRS)	○	○	○	○	○
Visual analog scale (VAS)	○	○	○	○	○
Wong-Baker faces pain scale	○	○	○	○	○
Other tools	○	○	○	○	○

Types of Physical Examination Assessments Used

Which of the following physical examination assessments do you use to evaluate pain? (select ALL that apply)

- ☐ Conditioned pain modulation
- ☐ Cutaneous mechanical pain sensitivity
- ☐ Dynamic mechanical cutaneous allodynia
- ☐ Mechanical detection threshold
- ☐ Pressure pain threshold
- ☐ Temporal summation
- ☐ Thermal pain threshold
- ☐ Vibration detection threshold
- ☐ Other
- ☐ None of the above

Please list what other tools you use to physically assess pain. __________________________________

How frequently do you use the following physical examination tools?

	NEVER	ALMOST NEVER	SOMETIMES	OFTEN	ALWAYS
Conditioned pain modulation	◯	◯	◯	◯	◯
Cutaneous mechanical pain sensitivity	◯	◯	◯	◯	◯
Dynamic mechanical cutaneous allodynia	◯	◯	◯	◯	◯
Mechanical detection threshold	◯	◯	◯	◯	◯
Pressure pain threshold	◯	◯	◯	◯	◯
Temporal summation	◯	◯	◯	◯	◯
Thermal pain threshold	◯	◯	◯	◯	◯
Vibration detection threshold	◯	◯	◯	◯	◯
Other tools	◯	◯	◯	◯	◯

Types of Questionnaires Used

Which of the following questionnaires do you use to evaluate pain? (select ALL that apply)

- ☐ Central sensitization inventory (CSI)
- ☐ Leeds assessment of neuropathic symptoms and signs (LANSS)
- ☐ Neuropathic pain questionnaire
- ☐ PainDETECT
- ☐ Pain diagram
- ☐ Pain sensitivity questionnaire
- ☐ Patient-reported outcomes measurement information system (PROMIS) pain interference
- ☐ Symptom severity scale
- ☐ Widespread pain index (WPI)
- ☐ Other
- ☐ None of the above

Please list what other questionnaires you use to evaluate pain.

____

How frequently do you use the following questionnaires?

	NEVER	ALMOST NEVER	SOMETIMES	OFTEN	ALWAYS
Central sensitization inventory (CSI)	○	○	○	○	○
Leeds assessment of neuropathic signs and symptoms (LANSS)	○	○	○	○	○
Neuropathic pain questionnaire	○	○	○	○	○
PainDETECT	○	○	○	○	○
Pain diagram	○	○	○	○	○
Patient-reported outcomes measurement information system (PROMIS) pain interference	○	○	○	○	○
Pain sensitivity questionnaire	○	○	○	○	○
Symptom severity scale	○	○	○	○	○
Widespread pain index (WPI)	○	○	○	○	○
Other tools	○	○	○	○	○

Additional Contributing Variables Assessed

Which of the following variables do you screen for in clinical patient interactions? (select ALL that apply)

- ☐ Anxiety
- ☐ Catastrophizing
- ☐ Depression
- ☐ Fear
- ☐ Sleep
- ☐ Stress
- ☐ Substance abuse
- ☐ Other
- ☐ None of the above

Please list what other contributing variables you screen for consistently in patient interactions.

____

How frequently do you screen for the following variables?

	NEVER	ALMOST NEVER	SOMETIMES	OFTEN	ALWAYS
Anxiety	○	○	○	○	○
Catastrophizing	○	○	○	○	○
Depression	○	○	○	○	○
Fear	○	○	○	○	○
Sleep	○	○	○	○	○
Stress	○	○	○	○	○
Substance abuse	○	○	○	○	○
Other	○	○	○	○	○

Part 3. Demographic Information

Which of the following most closely represent your preferred pronouns?

◯ She/her
◯ He/him
◯ They/them
◯ Other

How old are you (in years)?

____

How many years of experience do you have as a licensed physical therapist?

◯ 0-5
◯ 6-10
◯ 11-15
◯ 16-20
◯ 21-25
◯ >25

What is the highest academic degree you have completed?

◯ Bachelor's
◯ Master's
◯ Clinical doctorate (DPT)
◯ Academic doctorate (PhD, ScD, DSc, EdD, etc)

Have you completed an accredited post-professional residency program?

◯ Yes
◯ No

Have you completed an accredited post-professional fellowship program?

◯ Yes
◯ No

Are you a board-certified specialist in any of the following? (select ALL that apply)

☐ Cardiovascular and Pulmonary
☐ Clinical Electrophysiology
☐ Geriatrics
☐ Neurology
☐ Oncology
☐ Orthopedics
☐ Pediatrics
☐ Sports
☐ Women's Health
☐ None of the above

How would you best describe your primary professional role?

◯ Full-time administrator
◯ Full-time clinician
◯ Full-time educator
◯ Full-time researcher
◯ Other

You listed other for your primary professional role. How would you best describe your role?

____

The following questions are about your patient population. If you are not currently treating patients, please consider the proportion when you most recently were treating patients.

	0-25%	26-50%	51-75%	76-100%
Approximately what proportion of your patient population have a primarily peripheral body part related health condition? (e.g., patellofemoral pain, hamstring strain, lateral elbow tendinopathy, etc)	○	○	○	○
Approximately what proportion of your patients have a primarily spine related health condition? (e.g., low back pain, neck pain, temporomandibular joint disorder, etc)	○	○	○	○
Approximately what proportion of your patients would you classify as having chronic pain? (In this context, 'chronic pain' refers to pain that has lasted > 3 months.)	○	○	○	○
Approximately what proportion of your patients would you classify as having widespread pain? (e.g., fibromyalgia)	○	○	○	○
